# Insights into the Toxicity and Degradation Mechanisms of Imidacloprid Via Physicochemical and Microbial Approaches

**DOI:** 10.3390/toxics8030065

**Published:** 2020-09-01

**Authors:** Shimei Pang, Ziqiu Lin, Yuming Zhang, Wenping Zhang, Nasser Alansary, Sandhya Mishra, Pankaj Bhatt, Shaohua Chen

**Affiliations:** 1 State Key Laboratory for Conservation and Utilization of Subtropical Agro-Bioresources, Guangdong Province Key Laboratory of Microbial Signals and Disease Control, Integrative Microbiology Research Centre, South China Agricultural University, Guangzhou 510642, China; 20192047012@stu.scau.edu.cn (S.P.); 20192047010@stu.scau.edu.cn (Z.L.); 20193138058@stu.scau.edu.cn (Y.Z.); 20191047008@stu.scau.edu.cn (W.Z.); alansarynase@gmail.com (N.A.); sandhyamanshi@gmail.com (S.M.); pankajbhatt.bhatt472@gmail.com (P.B.); 2 Guangdong Laboratory for Lingnan Modern Agriculture, Guangzhou 510642, China

**Keywords:** imidacloprid, toxicity, microbial degradation, physicochemical degradation, degradation mechanisms

## Abstract

Imidacloprid is a neonicotinoid insecticide that has been widely used to control insect pests in agricultural fields for decades. It shows insecticidal activity mainly by blocking the normal conduction of the central nervous system in insects. However, in recent years, imidacloprid has been reported to be an emerging contaminant in all parts of the world, and has different toxic effects on a variety of non-target organisms, including human beings, due to its large-scale use. Hence, the removal of imidacloprid from the ecosystem has received widespread attention. Different remediation approaches have been studied to eliminate imidacloprid residues from the environment, such as oxidation, hydrolysis, adsorption, ultrasound, illumination, and biodegradation. In nature, microbial degradation is one of the most important processes controlling the fate of and transformation from imidacloprid use, and from an environmental point of view, it is the most promising means, as it is the most effective, least hazardous, and most environmentally friendly. To date, several imidacloprid-degrading microbes, including *Bacillus*, *Pseudoxanthomonas*, *Mycobacterium*, *Rhizobium*, *Rhodococcus*, and *Stenotrophomonas*, have been characterized for biodegradation. In addition, previous studies have found that many insects and microorganisms have developed resistance genes to and degradation enzymes of imidacloprid. Furthermore, the metabolites and degradation pathways of imidacloprid have been reported. However, reviews of the toxicity and degradation mechanisms of imidacloprid are rare. In this review, the toxicity and degradation mechanisms of imidacloprid are summarized in order to provide a theoretical and practical basis for the remediation of imidacloprid-contaminated environments.

## 1. Introduction

**Figure 1:**
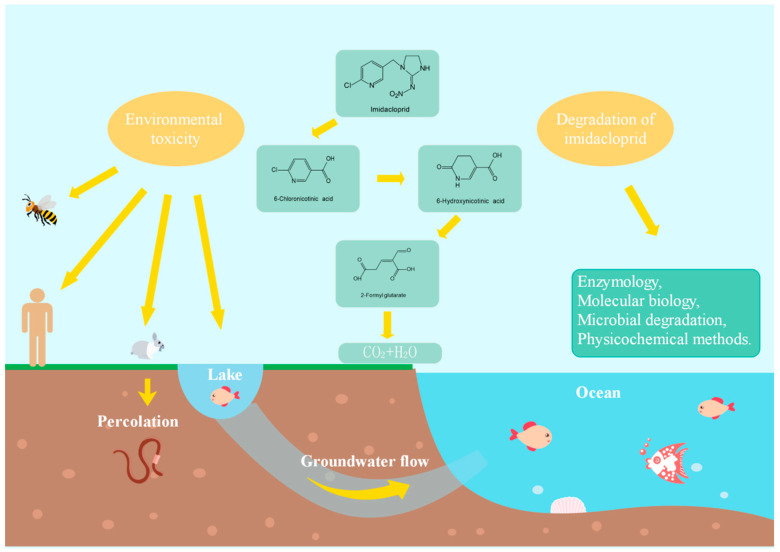
The fate and occurrence of imidacloprid in the environment.

Imidacloprid (1-((6-chloro-3-pyridinyl) methyl)-*N*-nitro-2-imidazolidnimine) is a colorless crystal neonicotinoid insecticide, which belongs to the chloronitroguanidine compounds, with a melting point of 143.8 °C. Imidacloprid is a polar compound, chemically stable under neutral and acidic conditions, but decomposes gradually in alkaline solutions [1]. Systemic insecticide neonicotinoids are recognized as a valuable tool for pest control, especially imidacloprid. It is widely used to control pests with piercing–sucking mouthparts on crops. In addition, it is reported that imidacloprid can effectively cure many cases of cardiopulmonary parasite infection in European feral cats [2]. Imidacloprid was first registered in the United States (U.S.) in 1992 by Miles Inc., and was approved by the U.S. Environmental Protection Agency (EPA) in 1994 [1]. Although imidacloprid was listed as one of the world’s best-selling pesticides in 2000, the European Union banned its use on outdoor crops entirely in 2018, after suspending its use in 2013 [3]. Imidacloprid can be used for leaf spraying and soil and seed treatment, all of which have had negative effects on ecosystem services, including the killing of non-target organisms with significant economic value, such as pollinators, honey providers, and natural insects that benefit farmers [4]. The pesticide toxicity load on the environment has increased approximately 50 times in the past 20 years. The negative influence on related ecosystems is the main cause of the increase in the toxicity load, which in turn may be a threat to the health of bees and other pollinators, resulting in a reduction in beneficial insect populations, other insects, and insect-eating birds [5]. Imidacloprid damages the biological nervous system through calcium ion imbalance, mitochondrial dysfunction, oxidative stress, and DNA damage, ultimately leading to biological death [6]. In recent years, imidacloprid has been reported to be an emerging contaminant in all parts of the world, and it has the potential to adversely impact ecosystems and human health (Figure 1). Therefore, the removal of imidacloprid residues from the ecosystem is a worldwide concern and priority.

**Figure 2:**
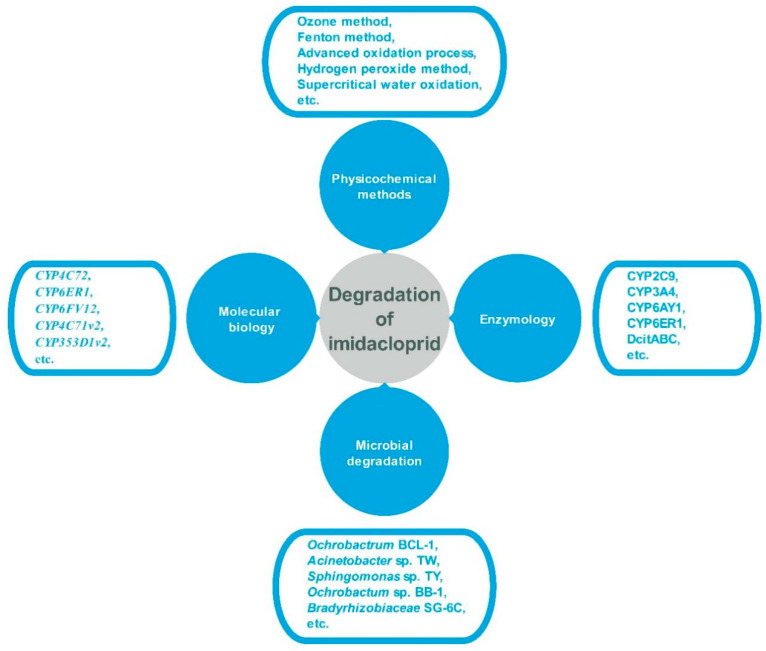
Degradation methods of imidacloprid.

In nature, (bio) degradation is one of the most important processes controlling the fate of and transformation from imidacloprid use. There are many aspects of pesticide degradation research worldwide [7,8,9,10]. The current main methods of imidacloprid degradation include: (1) Physical methods such as ultrasonic technology, washing, absorption, and ionizing radiation; (2) chemical methods such as hydrolysis, oxidative decomposition, and light chemical degradation; and (3) biological methods such as microbial degradation enzymes and engineering bacteria (Figure 2). These degradation-related studies have made considerable progress. Facing the persistence of surface water and the high leaching potential of groundwater, many traditional methods, such as Fenton oxidation, photodegradation, catalytic oxidation, adsorption, and biodegradation, are being used to deal with these problems [1].

The effects and fate of pesticides on soil depend, in part, on farming practices [11]. Pesticide residues in the soil are affected by the application of fertilizer, its pH value, the soil type, and the surface [12,13,14,15]. Under natural conditions, imidacloprid in soil or water can be degraded by microorganisms or through photolysis by sunlight (Figure 1). The efficiency of biodegradation is slower than that of photolysis, but it is not as easy to produce secondary pollution via this process. Currently, approximately 29 genes, 10 enzymes, and 18 bacteria are known to degrade imidacloprid efficiently. Most of the resistance genes and enzymes come from insects, while most of the bacteria are *Pseudomonas, Bacillus*, and *Aspergillus terreus* YESM3 (a fungus) [16]. However, few reviews have focused on the biochemical pathways and degradation mechanisms of imidacloprid. Therefore, the current work reviews the toxicity of imidacloprid to different organisms, the genes that can resist the toxicity of imidacloprid, and the microorganisms and their enzymes that can degrade imidacloprid, with the aim of establishing in-depth understanding of the degradation mechanisms of imidacloprid under different conditions.

## 2. Toxicity of Imidacloprid

With the increasing use of imidacloprid, the environmental and biological effects of imidacloprid have been investigated. There is a lot of evidence that imidacloprid has the potential to adversely impact ecosystems and human health. For instance, it was gradually discovered that imidacloprid is toxic to humans, and that imidacloprid is lethal to non-target organisms such as bats, earthworms, bees, fish, and shrimp (Table 1) [17,18,19,20,21].

### 2.1. Toxicity to Humans and Terrestrial Animals

Imidacloprid has relatively low toxicity for mammals, as compared to organochlorines and organophosphates [22]. However, with the popularity and increasing use of imidacloprid, its impact on non-target organisms and ecosystems is gradually becoming apparent. Imidacloprid is toxic to humans and it can lead to severe respiratory failure and a drop in consciousness [22]. Respiratory failure and loss of consciousness are the most serious but uncommon complications in acute toxicity cases. It has been shown that after spraying imidacloprid, the concentration of imidacloprid in the daily urine of farmers increased significantly and reached the highest concentration; the average recovery rate of imidacloprid and its main metabolite 6-chloronicotinic acid (6-CNA) in urine was 78.3–109.8%, and the quantitative limit was 0.029–0.038 ng·mL^−1^. In the same exposure environment, the concentration in women is typically higher than that of men, and younger children tend to have the highest exposure risk. Therefore, it is highly necessary to further study the effect of imidacloprid on humans [23,24]. The successful detection of new nicotinoids and their metabolites in recent years in a variety of human biological samples requires large-scale prospective studies to determine whether neonicotinoids pesticides have harmful effects on humans [17]. Young children and Asians may be more exposed to higher levels of imidacloprid than other age and ethnic groups, according to an analysis of the results from the 2015–2016 USA National Health and Nutrition Examination Survey (NHANES) [44]. Furthermore, imidacloprid was found in 36 urine samples taken from pregnant women living in the agricultural region of Almeria (Spain) [45]. Imidacloprid induces lymphocyte apoptosis and has shown the potential for DNA destruction in human lymphocytes and HepG2 cells [25]. The accidental inhalation of imidacloprid may result in severe gastrointestinal symptoms accompanied by respiratory distress and neuropsychiatric characteristics [26]. Imidacloprid metabolites are the main cause of imidacloprid toxicity, and changes in liver energy metabolism are closely related to its hepatotoxicity, which occurs when animals and humans are exposed to high concentrations of imidacloprid. However, imidacloprid induces liver fibrosis in quails by activating the TGF-β1/Smad pathway, and thus, TGF-β1/Smad may be a promising new treatment for liver fibrosis [27].

After two weeks of exposure to 1.0 mg·kg^−1^ of imidacloprid in rats, their cortisone and catecholamine levels changed and led to behavioral defects, especially in developing ones, whose mRNA levels of glucose transporters changed; the histopathological and immunohistochemical data showed that the pancreatic tissue structure was disordered, and the expression of insulin was decreased. Imidacloprid has also been shown to disrupt blood glucose regulation in developing and adult rats through hyperglycemic activity [18]. Imidacloprid has further been shown to interfere with the echolocation system of insect-eating bats; after imidacloprid poisoning, insect-eating bats may reduce the expression of auditory related proteins in the cochlea, the expression of sound-related FOXP2 in the superior colliculus, and the expression of auditory related otoferlin in the cochlea and lower colliculus, thus causing inflammation and cell apoptosis associated with mitochondrial dysfunction in the hippocampus and medial entorhinal cortex [19].

The earthworm (*Eisenia fetida*) is one of the most abundant species on land and maintains the ecological stability of soil. Imidacloprid is highly toxic to earthworms, with an LC_50_ of 3.05 mg·kg^−1^ and sublethal concentration of 2.0 mg·kg^−1^, as well as a mortality rate of 84.0%. After 14 days of imidacloprid exposure, significant destruction of earthworm epidermis and midgut tissues has been observed [21]. Meanwhile, imidacloprid and its metabolites are eliminated through the skin and excretion in Chinese lizards (*Eremias argus*) [28]. However, imidacloprid exposure inhibits thyroid hormone secretion in lizards, resulting in the down-regulation of thyroid hormone receptors and their associated metabolic enzyme genes [29]. Yang et al. first reported the endocrine disruption caused by imidacloprid in lizards; exposure to imidacloprid causes abnormal alignment of the spermatogenic epithelium, epididymal hyperplasia, and oligospermia in male lizards [30].

### 2.2. Toxicity to Aquatic Organisms

More and more pesticides are causing harmful effects on non-target species worldwide [23]. There are few studies on the effects of imidacloprid on vertebrates such as fish and species differences. Although imidacloprid is not suitable for use in water, it may enter water bodies through spray drift or runoff after use [1]. According to Annex VI of Regulation (EU) No. 528/2012 and Regulation (EC) No. 1272/2008, imidacloprid is classified as highly toxic to aquatic organisms, with a long duration of effects after ingestion [46]. The fate of imidacloprid in aquatic systems suggests that it is degraded by photolysis or microbial activity. Although imidacloprid has a fast photolysis rate, it still exists in the water column of the aquatic system [47]. Neonicotinoid concentrations can affect aquaculture populations [48], and imidacloprid has a sublethal effect on brown shrimp and zebrafish in the natural environment, especially in dehulling delay and growth reduction [20,31].

At sublethal levels, imidacloprid is associated with increased protein content and an unexpected reduction in imidacloprid-associated lipid peroxidation [49]. Compared to zebrafish, imidacloprid had a stronger effect on medaka in Japan, with the most significant effects being deformity damage and reduced growth [20]. Imidacloprid can be absorbed by the Sydney rock oyster and distributed in metabolically active tissues such as the adductor gills and digestive glands. The Sydney rock oyster has been shown to take four days to effectively remove imidacloprid from its tissues [50]. However, after exposure to imidacloprid for two weeks, oysters show a proportional increase in saturated fatty acids (SFAs), a change in both polyunsaturated fatty acid (PUFA) levels and the ratio of SFAs, and a change in both the level omega 3 fatty acids (n-3) and the proportion of omega 6 fatty acids (n-6), which significantly affect the biochemical processes and metabolites of oysters, which ultimately impact food quality and safety [32]. Imidacloprid alters the oxidative stress, cell detoxification, and multixenobiotic resistance (MXR) systems of Asian freshwater *Corbicula fluminea,* and the mRNA expression levels of its *Hsp* genes (i.e., *Hsp* 22, *Hsp* 40, *Hsp* 60, *Hsp* 70, and *Hsp* 90) and its genes related to the *M*XR (i.e., *abcb1* and *abcc1*) have been shown to be significantly down-regulated (*p* < 0.05) [33]. Imidacloprid has also been shown to have a greater adverse effect on the brain of juvenile Chinese rare minnows (*Gobiocypris rarus*) than on that of nitipyra. The organs most affected by imidacloprid in the neotropical fish *Prochilodus lineatus* are the liver and the kidneys, followed by the gills, which alters their biotransformation and the antioxidant enzyme activity in different tissues [34]. Imidacloprid alters the mitotic dynamics and causes aneugenic and clastogenic effects in the aquatic macrophyte *Bidens laevis* [35].

### 2.3. Toxicity to Insects

In insensitive insect species, toxic effects due to short-term exposure of less than 9 g·L^−1^ imidacloprid have become evident. However, it has been shown that when the stressor is stopped, the organisms recover without any long-term effects [51]. For the first time, researchers have documented the tritrophic movement of imidacloprid from plants to fungi to insects, resulting in a significant number of indirect deaths of non-target mycophasimilar beetles, and adversely affecting an important set of pathogen biological control pathways [52]. Compared to other non-target aquatic insects, neonicotinoid insecticides cause less harm to the early-life stages of the ramshorn snail and *Planorbella pilsbryi* [53]. The exposure of *Chironomus riparius* to sublethal concentrations for 10 days leads to an imbalance between reducing and oxidizing glutathione (GSH and GSSG), which slightly induces lipid peroxidation (malondialdehyde). Oxidative stress may be a related mechanism of neonicotinoid toxicity, reflected in the development and life cycle parameters of insects [36]. It has been shown that the sensitivity of the Asian tiger mosquito *Aedes albopictus* to imidacloprid decreases and epigenetic changes occur [37]. In greenhouse experiments, when imidacloprid was used at a concentration of 100% of the maximum field registration concentration (MFRC), the mortality rate for *Engytatus varians* was approximately 100% [38]. Imidacloprid can adjust the components of the desensitization and non-desensitization of nicotinic acetylcholine receptor (nAChRs), and it has a high affinity for the components of desensitization in American cockroach neurons [54].

Compared to the labeled concentration of imidacloprid, the median lethal dose (LC_50_) of imidacloprid on aphids is 6.4 × 10^−3^ times lower, and the effect of imidacloprid on aphids is lower than that of thiamethoxazole and sulfoxaflor [39]. After applying insecticide in the pupal stage, it was observed that imidacloprid has a negative effect on the mortality rate, sex ratio, body size, egg size, and sex ratio of the offspring of surviving wasps [55]. *Hippodamia variegata* is one of the most plentiful ladybug species in Greece, feeding on several aphids and other arthropods. Imidacloprid has been shown to decrease the longevity of adults under LC_10_ (3.92 mg·L^−1^) conditions and the survival rate under LC_30_ (8.69 mg·L^−1^) conditions. Sublethal concentrations of imidacloprid have a negative effect on lady beetles, which may reduce the biological control services they provide [40].

Neonicotinoid pesticides have led to a global decline in bee populations [19]. More and more research has documented the direct and indirect effects of imidacloprid insecticides on pollinators. For instance, *Apis mellifera* is viewed as a pointer of classic ecotoxicology tests, and imidacloprid is highly toxic to it [41]. Indeed, the large-scale use of pesticides is the main reason for the decline in bee populations; pesticides pose a major threat to bee biodiversity by damaging the maintenance of bee ecosystems and by reducing agricultural productivity [42]. For example, bee farms near agricultural areas have a high mortality rate during pesticide use [56]. Strong immune defenses are critical to bee health and colony survival, but adverse environmental factors may weaken the defenses of bees, making them more vulnerable to parasites and pathogens. Imidacloprid affects the individual immunity of bees, resulting in reduced disease resistance [43]. Although there is much evidence that neonicotinoids have a bad effect on bee behavior and colony growth, there are significant gaps in the knowledge regarding how exactly pesticides affect the health of pollinators [57]. However, new alternative insecticides are similarly deadly and harmful; thus, there may not be a good solution to distinguish useful insects from pests, but a reduction in the amount of pesticides released should be sought [58].

### 2.4. Synergistic Effects of Pesticides

It is well known that pesticide mixtures are used to manage pest resistance. Because the research results of single pesticide use are significantly different from that of mixtures, research on pesticide mixtures is of great significance. The different proportions of imidacloprid, acetochlor, and tebuconazole in mixtures also produces different types of acute toxicity to zebrafish. When imidacloprid, acetochlor, and tebuconazole were mixed in a ratio of 1:4:4, their toxicity was greater than that of a single pesticide on zebrafish [59]. The mixture of imidacloprid and cypermethrin encapsulates liposomes as nanocarriers, and a mixture of imidacloprid and cyhalothrin has been shown to be more effective in improving residues than their individual use alone [60]. Thus, the synergetic effects between pesticides cannot be ignored. Pyrethroid cypermethrin and imidacloprid alone or in combination can cause DNA damage in different tissues, as well as nuclear abnormalities in the red blood cells of neotropical freshwater teleost curimba *Prochilodus lineatus*. Pyrethroid cyhalothrin and imidacloprid mixtures also lead to an increase in the carbonylation of proteins and lipid peroxidation in the gills and liver after short exposure [61]. Imidacloprid, dichlorvos (DIC), and atrazine (ATZ) are three widely used commercial pesticides; their combination shows stronger toxicity, high lipid peroxidation, a decreased GSH content, and high antioxidant enzyme activity in all examined tissues, rather than selective sex organ toxicity [62]. It is important to study the combined toxicity of pesticides at low concentrations; for instance, the combined toxic effect of triazophos and imidacloprid on zebrafish (*Danio rerio*) is more pronounced with the expression of the 26 genes related to oxidative stress, cell apoptosis, immune system, and hypothalamic–pituitary–thyroid and hypothalamic–pituitary–gonadal axes at the mRNA level, and the embryos of zebrafish are more affected by the subsequent synergistic effects [63].

## 3. Degradation of Imidacloprid

With more and more imidacloprid accumulating in the environment, studying the optimal degradation method of imidacloprid is becoming more urgent. Remarkable discoveries have already been made in the physicochemical, molecular biological, enzyme, and microbial degradation of imidacloprid [64]. Indeed, in the face of the persistence of surface water and the high leaching potential of groundwater, many traditional physicochemical methods, such as Fenton oxidation, photodegradation, catalytic oxidation, and adsorption, are being used to deal with this problem [1]. Imidacloprid is quite stable in acidic and neutral water; however, under alkaline conditions, the hydrolysis of imidacloprid under different temperatures is enhanced by hydrolyzation, which follows quasi-first-order kinetics, but is relatively slow when the activation energy of the hydrolyzation is 42.72 KJ·mol^−1^, almost six times that of photodegradation [65]. Although pesticides can be completely degraded with the help of a catalyst, they are affected by various factors, such as initial concentration temperature and pH value, and the time of complete degradation varies. Pesticides are degraded by multiple hydroxylation reactions and aromatic ring breaking, generally resulting in the formation of non-absorbent intermediates such as carbon dioxide and other minerals [66].

### 3.1. Degradation of Imidacloprid by Physicochemical Methods

Chemical degradation of imidacloprid is mostly carried out by oxidation, mainly including the ozone, hydrogen peroxide, Fenton, and supercritical water oxidation methods (Table 2). It was recently found, for the first time, that imidacloprid in water can be effectively degraded in a short time by ozonation, and that the pH value affects the treatment while alkalinity inhibits oxidation in the degradation process [1]. Supported by superoxide radicals, imidacloprid is mainly degraded by hydroxyl radicals (OH). Due to the limitation of traditional treatment methods, an advanced oxidation process using a heterogeneous photocatalyst (AOP) has emerged [66], and imidacloprid has been shown to be almost completely degraded based on hydrodynamic cavitation. The time required for complete degradation of imidacloprid using hydrogen peroxide is 120 min, compared to 60 min for the Fenton process [67]. The treatment of imidacloprid with Fenton in a fluidized bed has been well studied; imidacloprid is almost completely removed, and both chemical oxygen demand (COD) and total organic carbon are consequently reduced [68]. Sodium percarbonate is non-toxic, easy to treat, and produces byproducts inherent in natural water matrix pesticide deoxidizers, a cleaner alternative to other oxidants in imidacloprid degradation [69]. The Ti/PbO_2_–Tb electrode exhibits higher oxygen evolution potential, lower charge transfer resistance, better stability, longer service life, and excellent electric catalytic activity—after two replications of an 8 mA·cm^−2^ current density, pH 9, temperature 30 °C, and 7.0 g·L^−1^ NaCl electrolyte, 76.07% of imidacloprid was shown to be removed in 5 h. In addition, the electrode showed admirable energy-saving performance and good repeatable use so it can effectively sewage [70]. Permanganate oxidation is a promising method to control imidacloprid contamination; its main reaction pathway is the hydroxylation of the C–H bond on the imidazole ring, thus reducing the toxicity of imidacloprid, which lays a foundation for the application of permanganate oxidation in the in-situ chemical remediation of imidacloprid [71]. Under simulated solar radiation, the Cu_2_ZnSnS4/TiO_2_ thin film heterostructure can degrade 57% of imidacloprid within 72 h. However, mineralization occurs slowly, and the removal efficiency of imidacloprid is less than 10% [72]. Based on a (SnO_2_)-Cu_2_ZnSnS_4_-TiO_2_ coating and methylene blue heteropair, the addition of Cu_2_ZnSnS_4_ extended to the activation domain of visible infra spectroscopy (VIS) samples, compared to the reference TiO_2_ film, can improve the removal of pollutants [73]. Additionally, molecularly imprinted titanium dioxide photocatalyst has been synthesized by the sol–gel method, and it has been shown that the degradation rate of insecticide by the impregnated catalyst is faster than that of bare titanium dioxide. Furthermore, in the impregnated catalyst, Ru/TiO_2_(1%) degrades three insecticides, namely imidacloprid, acetamiprid, and thiamethoxam, in the shortest time [66]. The higher the impregnation rate of nickel and ruthenium, the smaller the surface area of the titanium dioxide catalyst, and the more significant a difference in the band gap value. In other words, the catalytic efficiency can be improved by decreasing the band gap value.

The methods of physical degradation of imidacloprid include illumination, ultrasound, ionizing radiation, low-temperature plasma, ultra-high pressure, adsorption, washing, and heating. Polypyrrole, polyaniline, and sodium alginate composites with peanut husk have good adsorption performance and optimal influence on variables—such as pH, pesticide concentration, compound dose, contact time, and temperature—which can effectively remove imidacloprid, which can be used in the treatment of imidacloprid pesticide wastewater [64]. The catalytic activity of a semiconductor is affected by various parameters, such as crystal surface area, band gap, crystallinity, impurity, and density of surface hydroxyl. At room temperature and a pH of 10, the photocatalyst ZnO/CoFe_2_O_4_ magnetic nanocomposite at 0.05 g can completely degrade imidacloprid at 5 mg·L^−^^1^ by illumination for 45 min [74]. Metal-organic frameworks (MOFs) are mixed porous coordination polymers assembled by forming strong bridging bonds between organic ligands and metal ions. The combination of Fe_4_O_3_-GO-β-CD and MOFs can better improve the adsorption capacity and rate of nicotine; the adsorption capacity for imidacloprid at 100 mg·L^−1^ is 3.11 mg·g^−1^ [75].

The absorption spectra of imidacloprid in aqueous solutions are 211 nm and 268 nm, and the photodegradation of imidacloprid at the natural light wavelength (usually >300 nm) follows pseudo-first-order kinetics; the reaction rate increases with an increase of temperature and decreases with an increase in the initial concentration [65]. The photodegradation rate of neonicotinoids by monochromatic ultraviolet (UV) light (254 nm) is constant depending on the pH value, the properties of the specific compounds, and the presence or absence of free radical scavenging agents [76]. Photodegradation is the main degradation pathway of pesticides in water due to their structural characteristics, which directly affect the degradation dynamics and sustainability of pesticides, and may lead to two different phenomena, namely, photosensitization and photoquenching. Imidacloprid shows the strongest absorption value at a characteristic peak of 269 nm, with a good linear relationship (*R* > 0.99) between the concentration of imidacloprid and the absorbance, which could describe the degradation effect. The degradation rate of imidacloprid is 4.71% after 1 min of ultraviolet irradiation [77]. A modified carbon nitride/tungstophosphoric acid composite degradant of the imidacloprid rate constant (0.70/h) is the CN450 calcination at 450 °C (CN) for 6.4 times. Hydrogen radicals (·H) and OH are the main active substances in the visible light catalytic degradation process, which is a new kind of modified carbon nitride method combined with tungsten phosphoric acid [78]. Ultraviolet active (UVA) light can achieve more effective photodegradation than visible irradiation of imidacloprid [79]. Under the same conditions, the efficiency of the imidacloprid degradation in the UV-activated persulfate (UV/PS) system is higher than that of the UV-activated peroxymonosulfate (UV/PMS) system; the degradation of F^−^ and NO_3_^−^ in the UV/PS and UV/PMS systems has no obvious influence, while PO_4_^3−^ inhibits imidacloprid degradation in UV-PS system and enhances it in the UV-PMS system. UV/PS and UV/PMS may be potential technologies for removing imidacloprid from water treatment, and based on the total cost of electricity and chemicals, UV/PS is more cost-effective than UV/PMS. Usually, algal organic matter (AOM) can inhibit imidacloprid degradation [80]. The visible light-driven type III photocatalytic composite heterojunction H_3_PW_12_O_40_/TiO_2_-In_2_S_3_ was previously synthesized with the sol–gel method under visible light irradiation (*λ* = 400 nm), showing higher photocatalytic degradation activity (82.7%), thus performing better than H_3_PW_12_O_40_/TiO_2_ (26.7%), TiO_2_-In_2_S_3_ (20.6%), and titanium dioxide (TiO_2_) (16.0%). In addition, photogenic holes and ·OH are the main active substances in the degradation process [81]. The activity of sol–gel TiO_2_ is two–three times higher than that of the composite mixture of TiO_2_ and fly ash [82]. Bamboo vinegar can be used as an imidacloprid synergistic agent in agricultural production activities, mainly leading to a light quenching effect. The photodegradation rate decreases with an increase in the concentration of the vinegar under irradiation of a high-pressure mercury lamp [83]. In recent years, heterogeneous photocatalysis has emerged as a completely green environmental application technique. In particular, TiO_2_-based photocatalysis is currently considered to be an effective method for hydrogen production in the field of wastewater treatment and photocatalytic water cracking. However, the greatest disadvantage of photocatalysis is its non-selectivity [84].

The single oxidation process cannot completely remove imidacloprid in less than three hours, while double continuous processes, such as the electrolytic oxidation of ozone treatment, can. For quick removal of imidacloprid, it is proposed to use three or four orders to significantly improve the degradation and mineralization rate, through different advanced oxidation processes (including ozone oxidation, electrochemical oxidation, ultrasonic, ultraviolet radiation, and different combinations thereof) of the order [85]. The multi-stage intermittent adsorption system has a better adsorption effect than the single-stage adsorption system, which greatly reduces the cost of the process, especially biochar [86]. Nanomaterials also play an increasingly important role in physical adsorption. Silica nano hollow sphere (SNHS) and amino-functionalized silica nano hollow sphere (SNHS–NH_2_) adsorbents remove high concentrations of imidacloprid from aqueous solutions [87]. The nanoscale zero-valent iron/sodium persulfate system is an effective technique to control the water pollution caused by imidacloprid. A novel Zn_0.1_Cd_0.9_s/SnIn_4_S_8_ (ZCS/SIS) core-shell nanorod photocatalyst can promote photoinduced charge separation and transfer. Compared to the original Zn_x_Cd_1__−x_S (ZCS) and SnIn_4_S_8_ (SIS), the core-shell composite shows significantly improved photocatalytic activity. In a previous study of ZCS/SIS, 55% of imidacloprid was degraded within 240 min, and the apparent rate constant was 16.9 times higher than that of pure ZCS [88]. Ungstophosphoric acid (HPW) and acidified carbon nitride (ACN), through hydrogen bonding, interact to form a new efficient mixed photocatalyst. The photocatalytic activity of HPW/ACN composites can be improved by improving the hole-electron separation efficiency. In another study, under visible light exposure (*λ* > 400 nm), the photocatalytic degradation rate constant of HPW/ACN was shown to be 16 times higher than that of ACN imidacloprid [89]. GO/Fe_3_O_4_/TiO_2_-NiO can be used as an appropriate photocatalyst for the degradation of organophosphorus pesticides in visible light. At pH 9, 0.08 g of GO/Fe_3_O_4_/TiO_2_-NiO can degrade at least 5 mg·L^−1^ pesticides [90]. Zinc-based materials and an enzyme hybrid system are considered to be effective catalysts for the treatment of refractory pollutants. In wastewater, using attenuated total reflection Fourier transform infrared spectroscopy (ATR–FTIR), Ce-doped ZnO oxide photocatalyst, which is included in the poly(styrene-co-maleic anhydride) (SMA) nanofiber and is combined with the active enzyme soybean peroxidase (SBP), also shows high efficiency and may be used to eliminate refractory pollutants in water treatments. These supported treatment systems potentially increase the use of self-supporting materials in reducing contaminants [91]. Cork boiling wastewater (CBW) contains a large number of polyphenol compounds, which can form ferric and ferrous compounds, helping to reduce ferric iron and to promote iron availability and regeneration. At pH 5, the mineralization rate of imidacloprid is greater than 70%, which is similar to that of pH 3 without CBW. The observed effect of reusing gangue is similar to that of commercial iron-chelating agents such as ethylenediamine-*N*,*N*-disuccinic acid (EDDS) added to the Fenton method [92]. The use of Fe (III)–EDDS in the treatment of highly contaminated imidacloprid wastewater with a near-neutral pH has both advantages and limitations. The photolysis of Fe (III)-EDDS produces free radicals independent of the presence of H_2_O_2_, and the toxicity of the initial complex and the intermediates produced by its photolysis is zero. However, not all of the compounds are completely degradable, and the addition of more and more EDDS is needed for continuous processing, leading to a degradation percentage of approximately 30% [93]. Ag_2_O nanoparticles can be evenly distributed on the surface of g-C_3_N_4_ to form the heterogeneous structure of Ag_2_O/g-C_3_N_4_. The synthesized heterojunction photocatalyst has a good broad-spectrum response and can effectively utilize solar energy. A recent study showed that after imidacloprid was removed by Ag_2_O/g-C_3_N_4_ and exposed to visible light for 30 min and near-infrared light for 120 min, the removal rate of total organic carbon reached high mineralization rates of 62.7% and 50.6%, respectively. Thus, the Ag_2_O/g-C_3_N_4_ heterojunction can be used as an efficient visible and near-infrared photocatalyst [94,95].

**Figure 3:**
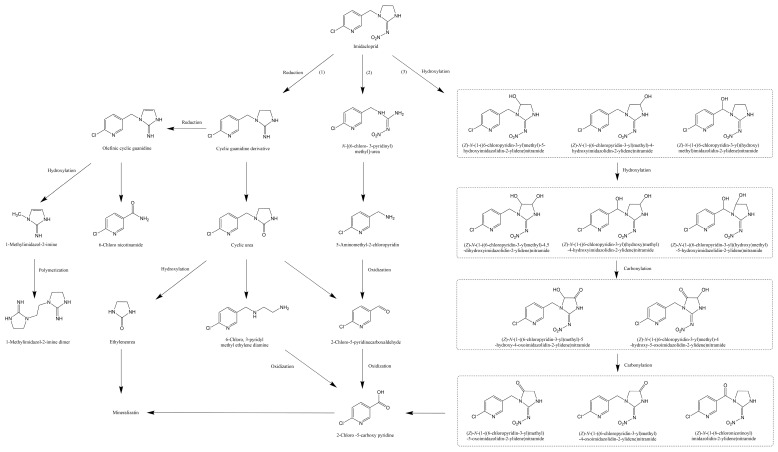
The physicochemical degradation pathways of imidacloprid, adapted from [96,98,101].

The physicochemical degradation pathways of imidacloprid are comprehensively collected here. Based on previous research, the degradation pathway of imidacloprid in physicochemical degradation is summarized. Many products are produced during the degradation of imidacloprid, and almost all of the byproducts contain the complete chloro-pyridine ring. As shown in Figure 3, the degradation of imidacloprid is predominantly divided into four main directions. In pathway (1), reduction plays a primary role, which facilitates the removal of nitroso and the generation of C=N. Under the reduction, imidacloprid is gradually transformed to cyclic guanidine derivatives and olefinic cyclic guanidine. Olefinic cyclic guanidine further generates 1-methylimidazol-2-imine, which produces the 1-methylimidazol-2-imine dimer through polymerization. At the same time, the cyclic guanidine derivatives form cyclic urea, which eventually produces ethyleneurea and 2-chloro-5-carboxy pyridine. In pathway (2), the breaking of the C–N bond of the tertiary amine and the *N*-dealkylation of the amine on the imidazolidine ring play very important roles, which lead to the generation of *N*-[(6-chloro-3-pyridinyl) methyl]-ethyl and 5-aminomethyl-2-chloropyridin. Subsequently, 5-aminomethyl-2-chloropyridin is oxidized to become the primary intermediate 2-chloro-5-carboxy pyridine, which is eventually mineralized. In pathway (3), complete degradation does not occur. Based on the related products, it can be inferred that imidacloprid is degraded to produce 2-chloro-5-carboxy pyridine by hydroxylation and carbonylation, respectively. At the end of the three pathways, the pyridine ring undergoes dechlorination, and the final mineralization of imidacloprid is achieved [96,97,98,99,100,101].

### 3.2. Degradation of Imidacloprid by Microbial Strains

For pesticides remaining in the soil, microbial degradation is the most important way to degrade them, and the degradation rate depends on pesticide type, soil moisture content, redox status, and soil microorganisms [102,103,104,105]. The advantages of biodegradation are (1) its strong selectivity; (2) its safety to people, livestock, crops, and the natural environment; (3) it does not harm natural enemies; and (4) it does not easily produce resistance [106,107,108,109,110]. Microbial degradation is a clean, efficient, and ecologically friendly method for the remediation of organic compounds in soil and water without secondary pollution [11,111,112,113]. As such, several imidacloprid-degrading strains have been isolated and characterized (Table 3). The biodegradation of imidacloprid by different types of bacteria was first reported in 2007 [114], when it was shown that the *Leifsonia* strain PC-21 co-metabolized imidacloprid and degraded 37–58% of 25 g·L^−1^ of imidacloprid in a trypsin solution containing 1 g·L^−1^ succinic acid and d-glucose in 3 weeks at 27 °C [114]. The increasing use of potentially dangerous chemicals in agriculture has led to increased concern for the environment and public health, but the use of biological control strategies is a risk-free and economically viable approach [115,116,117,118,119]. Soil organic matter is the main adsorption medium of imidacloprid, and the microorganisms in the soil can effectively degrade imidacloprid. Indeed, the effective strain is an effective method for bioremediation of imidacloprid-contaminated soil.

*Pseudomonas* has been found to have the highest effect of all bacteria with regard to imidacloprid degradation. *Pseudomonas* sp. PRT 52 can be isolated from fields by soil enrichment, and is able to metabolize three different chlorinated pesticides, namely, imidacloprid, endosulfan, and coragen. Using imidacloprid as the only carbon source, it was shown that this biological strain is able to degrade 46.5% of 0.5 mm of imidacloprid in 40 h following first-order kinetics [120]. *Rhodopseudomonas capsulata* is a type of archaea. The addition of wastewater containing *R. capsulata* can effectively remove imidacloprid, improve soil fertility, and improve microbial community structure. The remaining organic matter in the wastewater provides enough carbon and energy for algae. The main difference between *R. capsulata* and other microorganisms is that it has many complex metabolic pathways, with both active and dormant major metabolic functions. Dormant metabolic pathways need to be activated by signal transduction pathways. In a previous study, the *CPM* gene was expressed to synthesize P450 after the *R. capsulata* enters the wastewater for 24 h; when the concentration of imidacloprid was 1000 mg·L^−1^, the growth rate of *R. capsulata* and the removal rate of imidacloprid were the best—97% of imidacloprid could be removed in 5 days. *R. capsulata* does not cause secondary pollution to the environment [121], and *Pseudomonas* sp. 1G is capable of converting imidacloprid into urea metabolites and denitrification products [122]. Under the cooperative metabolism mechanism of *Pseudoxanthomonas indica* CGMCC 6648, in the presence of glucose, imidacloprid can be degraded to 5-hydroxy imidacloprid and olefin imidacloprid in six days; in the presence of lactose, imidacloprid can be degraded to 5-hydroxy imidacloprid in 48 h; and imidacloprid in the presence of pyruvate can form olefin imidacloprid in 96 h. *P. indica* CGMCC 6648 can rapidly degrade imidacloprid and form olefin imidacloprid. Since olefin imidacloprid is more toxic, the amount of imidacloprid required can be reduced [123].

Microbes isolated from nature can adapt to a wider range of pH and temperature values, making it easier to generalize in the environment [111,124,125]. *Sphingomonas* sp. TY and *Acinetobacter* sp. TW, which are isolated from tobacco waste, can reduce imidacloprid and acetamprid in a wide range of pH and temperature values, and can also degrade nicotine pesticides [126]. A strain of *Bacillus thuringiensis* isolated from contaminated marine sediments is able to degrade 71% of imidacloprid in 11 days [127]. One of the most promising biological control agents is *Trichoderma*, which is widely distributed in different agricultural climates and is ubiquitous in soil and root ecosystems. With the characteristics of different types of biological controls and promoting plant growth, it is a powerful biological control weapon that can combat a variety of plant pathogens and can provide protection for sustainable production and management in agriculture [128]. Separated from agricultural soil, *Klebsiella pneumonia* strain BHC1 degrades 78% of imidacloprid within 7 days at 30 °C, and metabolites such as nitroguanidine, imidacloprid guanidine, and 6-chloronicotinic acid can be obtained [129].

Under optimal conditions, microorganisms screened by enrichment culture technology can achieve an optimal degradation effect. Under autoclaved and non-autoclaved conditions, *Bacillus aerophilus* and *Bacillus alkalinitrilicus* degrade more than 90% of imidacloprid in clay loam within 56 days. The metabolites of imidacloprid are 6-chloronicotinic acid, nitrosimine, and imidacloprid-NTG, which are not affected by sterilization. It has been shown that in both groups, the dissipative pattern after imidacloprid inoculation does not conform to first-order kinetics, and the half-life of the bacteria is 13–16 days [130]. Through high-performance liquid chromatography (HPLC) and liquid chromatography–mass spectrometry (LC–MS) studies, *Bradyrhizobiaceae* strain SG-6C hydrolyzes imidacloprid to dechlorinated 6-chloronicotinic acid and then 6-hydroxynicotinic acid, which is then further metabolized by the nicotinic acid pathway. 6-CNA can be used as the only carbon source, and 20 mg·L^−1^ (0.1 mM) of 6-CNA can be completely degraded in 1% (v/v) seed culture within 152 h [131]. By contrast, *Aspergillus terreus* YESM3 degrades imidacloprid faster and more efficiently than *Leifsonia* strain PC-21 [16]. Under the same mechanism as human cytochrome P450, *Streptococcus maltophagus* CGMCC 1.1788 requires the use of sucrose as an energy source to provide NADPH (NADH) for continuous enzymatic biocatalysis, where the optimal conditions for the reaction are 30 °C and pH 7.2 [132]. *Mycobacterium* sp. strain MK6 has been identified to completely convert imidacloprid to 6-chloronicotinic acid, but there is no evidence of CO_2_ mineralization during culture [133].

The single microbiological dosage form, the backward production technology, the unstable physicochemical index, and the effective ingredient content of the product have formed a bottleneck in the development of microbiological pesticides. The empirical method for optimizing process parameters is complex and expensive, without considering the effect of parameter interaction. In contrast, artificial neural networks (ANNs) and the response surface method (RSM) are effective methods for modeling and optimizing processes, particularly those in environmental systems. The combination of modeling tools and experimental methods reduces laboratory research, saves time and money, and can be extended in further research [69].

### 3.3. Molecular Biology of Imidacloprid Degradation

The recently discovered imidacloprid degradation and resistance genes are reviewed herein (Table 4). Three kinds of enzymes in the epidermal protein (CP), cytochrome P450 mono-oxygenase (P450), and glutathione synthase (GSS) family encode imidacloprid resistance [139]. Cytochrome P450 is widely found in nature and plays an important role in many biological processes, including hormone synthesis and the metabolism of exogenous compounds. Cytochrome P450 is thought to provide resistance to pesticides by degrading exogenous compounds into more soluble and less toxic forms [140]. The P450 regulatory gene (*cpm* gene) was first expressed in the *R. capsulata* to control the synthesis of P450 to remove imidacloprid [121].

A new P450 gene, *CYP6FV12*, has been discovered in *Bradysia odoriphaga*. The cDNA sequence is 2520 bp long and its open reading frame (ORF) encodes 519 amino acids. According to real-time quantitative polymerase chain reaction (PCR), *CYP6FV12* is the most expressed in the fourth instar larvae, which is 154.32 times higher than in eggs. When the concentration of imidacloprid increases, and the expression of *CYP6FV12* also increases, and when this gene is knocked out, mortality increases by 28.62 [97]. Anti-imidacloprid Colorado potato beetle up-regulates the common cytochrome P450 gene (*CYP6BJ^a/b^*, *CYP6BJ1v1*, *CYP9Z25*, and CYP9Z29) by using the metabolic resistance of the exogenous transcription factors CncC and Maf to achieve the metabolic detoxification of potato plant allelopathic and imidacloprid [141]. Whitefly, one of the most serious pests controlled by imidacloprid, has developed imidacloprid resistance mainly through the overexpression of *CYP6CM1*. The *CYP6CM1* variant has insufficient metabolic activity against dinotefuran and does not cause cross-resistance or a low cross-resistance level [142]. Cross-resistant *CYP6ER1* mutants of imidacloprid-resistant *Nilaparvata lugens* against other neonicotinoids are present. With substrate selectivity, the resistance level of *N. Lugens* to dinotefuran is low [143]. *CYP353D1v2* is related to the short-term selection of imidacloprid resistance, and the overexpression of *CYP4C71v2, CYP4C72,* and *CYP6AY3v2* is related to the long-term selection of imidacloprid resistance. *Bacillus striatum* containing these genes has a strong resistance to imidacloprid, and after the deletion of these genes, it becomes sensitive to imidacloprid [144]. In Chinese *Bemisia tabaci*, the four genes *CYP4CS3*, *CYP6CX5*, *CYP6DW2*, and *CYP6CM1* show cross-resistance to imidacloprid and acetamiprid, among which *CYP6CM1* is the most frequently expressed [145].

Citrus greening disease, otherwise known as huanglongbing disease, is caused by Candidatus *Liberibacter asiaticus*. The use of insecticide to control *Diaphorina citri* has become the most important means to control huanglongbing disease. The *AChE* and *ChE* genes in *D. citri* control the resistance of *D. citri* to imidacloprid. Removing these two genes with RNA interference from acetylcholinesterase makes *D. citri* more sensitive to pesticide [146]. *Nlhsp70*, the heat shock protein (Hsp) of *N. lugens*, is an important gene of resistance to imidacloprid stress, and its characterization showed a full length of 2805 bp with an ORF of 1896 bp, which has a high homology with other species. *Nlhsp70* can withstand heat, while in a cold environment, its expression is decreased [147]. nAChRs is the molecular target of imidacloprid, and mutations in nAChRs can produce high resistance. In the western flower thrips, *Frankliniella occidentalis* polybactericidal resistance is positively correlated with the truncated Foccα6 transcript. The percentage of Foccα6 can be used as a diagnostic tool to detect and quantify multibactericidal resistance in the field [148]. Four detoxifying genes of *Sitobion avenae* fabricius, namely, *CYP6a14*-1, *CYP307A1*, *gst1-1-1*, and *COE2*, have been shown to increase resistance to imidacloprid by RNA interference (RNAi) feeding. These genes can be used as silent targets for field populations with high resistance to imidacloprid [149]. *cch2* is a candidate gene encoding the initial dechlorination step, and is a member of the metal-dependent hydrolase superfamily, similar to the TRZ/TZ family of chlorotic enzymes. It is mobilized into the *Bradyrhizobiaceae* strain SG-6C by binding with an integrative and conjugative element (ICE) that introduces 6-hydroxynicotinic acid into the existing nicotinic acid mineralization pathway [131]. UGT-mediated (UDP-glycosyltransferase) insecticide resistance to insects is less reported than that of other detoxifying enzymes such as P450s, esterases (ESTs), and glutathione *S*-transferases (GSTs); some raise the *D. citri* resistance of the UGT gene to imidacloprid, for example, the *UGT375A1 UGT383A1, UGT383B1*, and *UGT384A1* genes can significantly improve the group resistance to imidacloprid. Silencing these genes significantly increases the sensitivity of the population to imidacloprid [150].

### 3.4. Enzymology of Imidacloprid Degradation

The recent discoveries of imidacloprid degrading enzymes were reviewed, as shown in Table 5. Detoxification of pesticides by insects depends, in part, on the ABC transporter (detoxification of xenobiotics), which helps to detoxify heterologous organisms. The anti-imidacloprid gene *ABCG3* is derived from *Bemisia tabaci*, and its expression level in females is higher than that in males. Inhibiting ABCG3 with RNA interference can significantly increase the mortality rate of adult *Bemisia tabaci* [151]. Forty-four DcitABC transporters have been identified from the *D. citri* transcriptome and genomic database. DcitABC transporters are highly expressed in multiple *D. citri* sites, and they participate in the detoxification of imidacloprid, together with metabolic enzyme genes [152].

Bioinformatics analysis has demonstrated that BtCPR is a transmembrane protein. It contains conservative binding domains (FAD, FMN, and NADPH) and has a molecular weight (MW) of 76.73 kDa. The highest levels of BtCPR occur in the head tissue and in the adult male whitefly. The sensitivity of BtCPR to imidacloprid is significantly increased when it is inhibited [153]. Furthermore, there is no cross-resistance between imidacloprid and pyrazine in *N. lugens*. In *N. lugens*, two P450s, namely, CYP6AY1 and CYP6ER1, can effectively metabolize imidacloprid and cause resistance in *N. lugens* [154]. In the Chinese lizard (*Eremias argus*), aldehyde oxidase (AOX) and CYP2C9 are key enzymes in in nitro reduction, and CYP3A4 controls the hydroxylation and desaturation processes [50].

**Figure 4:**
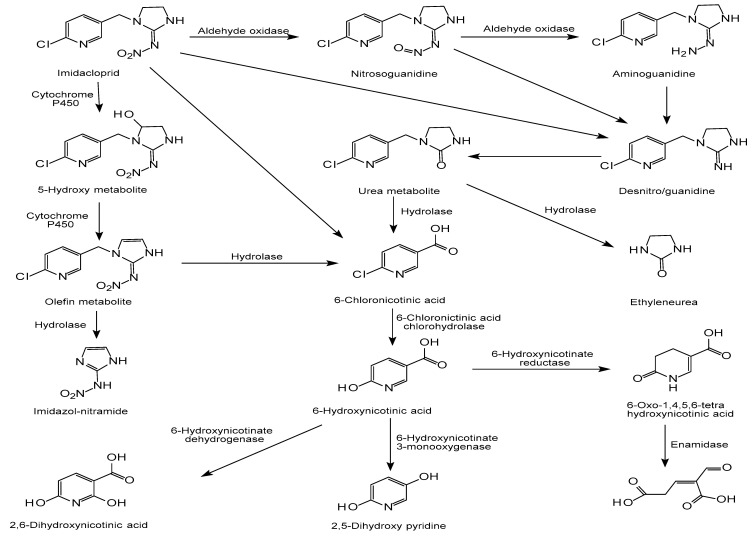
The biodegradation pathways of imidacloprid, adapted from [121,122,130,137].

The R81T mutant, derived from *Myzus persicae* and aphid cotton, is a mutant of the nicotinic acetylcholine receptor (acetyl) β1 subunit and a is key determinant of the binding of neonicotinic insecticides to nAChR. The R81T mutation has high resistance to imidacloprid, and has different effects on cyano-nitrosubstituted neonicotinoids and sulfoniloxalic acid [140]. In vitro, AOX metabolizes many exogenous pesticides, and both it and cytochrome P450 (CYP) metabolize imidacloprid. However, in New Zealand rabbits, the AOX metabolic pathway plays a more important lethal role in the systemic toxicity of imidacloprid [31]. All of the possible pathways of imidacloprid biodegradation are comprehensively collected here. The degradation mechanism of imidacloprid in living organisms has been preliminarily studied and the enzymes involved in the degradation pathway include Cytochrome P450, cytosolic AOX, and Enamidase [131,155]. There are three main biological degradation pathways of imidacloprid, namely, hydroxylation of the imidazolidine ring, reduction of the nitro group, and loss of the nitro group, as shown in Figure 4. In the first pathway, imidacloprid is formed into olefin imidacloprid by hydroxylation and dehydration reactions. Cytochrome P450 is a key family of enzymes in the imidacloprid hydroxylation pathway, and some isoenzymes are selective for imidacloprid catalysis [156]. In the second pathway, imidacloprid is gradually reduced to nitrosoguanidine and aminoguanidine, in which AOX plays a very important role [155]. In the third pathway, imidacloprid is suspected to lose the nitro group directly, but the related enzyme has not yet been isolated and identified. The same metabolites, namely, 6-chloronicotinic acid and 6-hydroxynicotinic acid, are produced in all three pathways. Interestingly, 6-hydroxynicotinic acid also has three different metabolic pathways under the action of its corresponding enzyme, producing 2,6-dihydroxynicotinic acid, 2,5-dihydroxy pyridine, and 2-formyl glutarate, respectively. By the end, the products of all three pathways are eventually mineralized. However, the molecular mechanisms of imidacloprid degradation still need more exploration and further improvement.

## 4. Conclusions and Future Prospects

Imidacloprid has been widely used because of its relatively low toxicity and highly efficient insecticidal activities. However, with the accumulation of imidacloprid in the natural environment, its toxic effects on non-target organisms cannot be ignored. Imidacloprid is toxic to mammals including human beings, and it can directly kill bats, earthworms, bees, fish, shrimp, and other non-target organisms. Hence, the development of a quick, easily applied, cost-effective, and eco-friendly method against imidacloprid is necessary. In recent years, many methods, mainly including physicochemical degradation and biodegradation, have been developed and applied for the remediation of imidacloprid from the environment. Physicochemical degradation methods have high degradation speed, strong targeting, and low operational requirements, and they are easy to control and result in complete degradation. However, they have poor stability, a narrow application scope, and a high cost, and they can easily cause secondary pollution. Biodegradation is characterized by (1) its strong selectivity; (2) its safety to human beings, livestock, crops, and the natural environment; (3) it does not harm natural enemies; and (4) it causes no resistance. Compared with physicochemical methods, biodegradation is considered to be the most promising approach. Microbes have the potential to detoxify the xenobiotic compounds easily through various metabolic pathways. However, published literature about the use of microbes to remediate the imidacloprid-contaminated environment is still insufficient. In addition, the utilization of degrading genes/enzymes and the development of engineered degrading microorganisms are needed. Furthermore, we need to investigate whether the synergistic degradation of microbial consortia is helpful to prevent the accumulation of toxic metabolites. Recently, omics-based technologies provide new insights into pesticide biodegradation, which may help to make an efficient strategy for bioremediation of imidacloprid-contaminated environment.

## Figures and Tables

**Table 1:** Toxicity of imidacloprid.

S. NO.	Sources	Imidacloprid Concentration/Median Lethal Concentration (LC_50_)/Median Lethal Dose (LD_50_)	Results	References
1.	*Podisus nigrispinus*	LC_50_ = 3.75 mg·L^−1^	Histological changes are observed in the mesenteric epithelium	[22]
2.	Humans	0.05 mg·L^−1^	Leads to severe respiratory failure and a drop in consciousness, induces lymphocyte apoptosis	[23,24,25,26,27]
3.	Rats	1.0 mg·kg^−1^	Disorders the pancreatic tissue structure, decreases the expression of insulin, and disrupts blood glucose regulation	[18]
4.	Insect-eating bats	LD_50_ = 115 mg·kg^−1^	Interferes with the echolocation system, reduces the expression of auditory-related proteins in the cochlea	[19]
5.	Earthworm (*Eisenia fetida*)	LD_50_ = 3.05 mg·kg^−1^	Inhibits fecundity and cellulase activity, and damages the epidermal and midgut cells of earthworm	[21]
6.	Lizards (*Eremias argus*)	20 mg·kg^−1^	Inhibits thyroid hormone secretion, causes abnormal alignment of the spermatogenic epithelium, epididymal hyperplasia, and oligospermia in male lizards	[28,29,30]
7.	Brown shrimp	0.5 g·L^−1^	Sublethal effect, especially in dehulling delay and growth reduction	[20,31]
8.	Zebrafish (*Danio rerio*)	400 µg·L^−1^	Sublethal effect, especially in dehulling delay and growth reduction	[20]
9.	Medaka (Japanese rice fish)	0.2 µg·L^−1^	Causes deformity and reduces growth	[20]
10.	Sydney rock oyster	2 mg·L^−1^	Affects the biochemical processes and metabolites, such as the parotid and digestive glands and other metabolic active tissue	[32]
11.	Asian freshwater (*Corbicula fluminea*)	2000 µg·L^−1^	Causes oxidative stress, cell detoxification, and multixenobiotic resistance systems	[33]
12.	Neotropical fish (*Prochilodus lineatus*)	5.45–359 µg·L^−1^	Alters the biotransformation and the antioxidant enzyme activity in different tissues	[34]
13.	Aquatic macrophyte (*Bidens laevis)*	1–1000 µg·L^−1^	Alters the mitotic dynamics and causes aneugenic and clastogenic effects	[35]
14.	*Chironomus riparius*	LC_50_ = 31.5 mg·L^−1^	Leads to an imbalance between reducing and oxidizing glutathione	[36]
15.	Asian tiger mosquito (*Aedes albopictus*)	LC_50_ = 47.5 µg·L^−1^	Decreases the sensitivity to imidacloprid	[37]
16.	*Engytatus varians*	1400 mg·L^−1^	Dies at a concentration of 100% of the maximum field registration concentration	[38]
17.	Aphids	LC_50_ = 3.186 mg·L^−1^	The LC_50_ of imidacloprid is 6.4 × 10^−3^ times lower	[39]
18.	*Hippodamia variegata*	LC_10_ = 3.92 mg·L^−1^; LC_30_ = 8.69 mg·L^−1^	Decreases adult longevity and survival rate	[40]
19.	*Apis mellifera*	LD_5048h_ = 0.0037 lg/bee	Poses a major threat to bee biodiversity, affects the individual immunity of bees	[41,42,43]

**Table 2:** The physicochemical methods for the degradation of imidacloprid.

S. NO.	Physicochemical Methods	Reaction Conditions	Results	References
1.	The photocatalyst ZnO/CoFe_2_O_4_ magnetic nanocomposite	Room temperature and pH of 10	0.05 g of the photocatalyst can completely degrade the concentration of imidacloprid at 5 mg·L^−^^1^ for 45 min	[74]
2.	Based on (SnO_2_)-Cu_2_ZnSnS_4_-TiO_2_ coating heteropair methylene blue	Visible infra spectroscopy (VIS)	Improves the removal of imidacloprid (10 mg·L^−^^1^) compared to the reference TiO_2_ film	[73]
3.	Ru/TiO_2_	Molecularly imprinted titanium dioxide photocatalyst synthesized by the sol–gel method	9.5 × 10^−^^5^ M of imidacloprid is completely degraded in 300 min	[66]
4.	Bamboo vinegar	Under the irradiation of a high-pressure mercury lamp	The half-lives of imidacloprid is 17.6 min at a concentration of 5 mg·L^−^^1^	[83]
5.	A modified carbon nitride/tungstophosphoric acid composite	-	The degradation rate constant(0.70 h^−1^) of imidacloprid (10 mg·L^−^^1^) is 6.4 times than that of carbon nitride 450	[78]
6.	UV-activated persulfate (UV/PS) and UV-activated peroxymonosulfate (UV/PMS)	UV	UV/PS and UV/PMS systems achieve great removal efficiency of imidacloprid (2.5 mg·L^−^^1^)	[80]
7.	Type III photocatalytic composite heterojunction H_3_PW_12_O_40_/TiO_2_-In_2_S_3_	Under visible light irradiation (*λ* = 400 nm)	Higher photocatalytic degradation activity (82.7%)	[81]
8.	Double continuous process	The electrolytic oxidation of ozone treatment	Completely removes imidacloprid (5 mg·L^−^^1^) in 120 min	[85]
9.	Heterogeneous structure of Ag_2_O/g-C_3_N_4_	Visible light for 30 min and near-infrared light for 120 min	The degradation rate of imidacloprid (10 mg·L^−^^1^) is 66% in 120 min	[94]
10.	Fe(III)-ethylenediamine-*N*,*N*-disuccinic acid (EDDS)	Solar radiation	Removes more than 90% of imidacloprid (60 mg·L^−^^1^)	[93]
11.	Cork boiling wastewater	pH 5	Removes more than 95% of imidacloprid (70 mg·L^−^^1^)	[92]
12.	Combined advanced oxidation process based on hydrodynamic cavitation	Hydrogen peroxide	Completely removes imidacloprid (60 mg·L^−^^1^) in 120 min	[67]
13.	Combined advanced oxidation process based on hydrodynamic cavitation	The Fenton process	Completely removes imidacloprid (60 mg·L^−^^1^) in 60 min	[67]
14.	Zinc-based materials and an enzyme hybrid system	Using attenuated total reflection Fourier transform infrared spectroscopy	Degrades 85% of imidacloprid (10 mg·L^−^^1^) within 24 h	[91]
15.	GO/Fe_3_O_4_/TiO_2_-NiO	At pH = 9, in visible light	0.08 g can catalyze 97.34% of imidacloprid (10 mg·L^−^^1^) in 30 min	[90]
16.	Tungstophosphoric acid and acidified carbon nitride	Under visible light exposure (*λ* > 400 nm)	Tungstophosphoric acid and acidified carbon nitride imidacloprid is degraded 16 times higher than that of acidified carbon nitride (ACN) imidacloprid	[89]
17.	Zn0.1Cd0.9s/SnIn_4_S_8_	In visible light	55% imidacloprid (5 mg·L^−^^1^) is degraded within 240 min	[88]
18.	Solar heating technology	Solarization and biosolarization	The disappearance rate of imidacloprid is increased	[95]
19.	Irradiation	UV	The degradation rate of imidacloprid (0.0255 mg·mL^−^^1^) is 98.43% after ultraviolet irradiation for 10 min	[77]
20.	Permanganate oxidation	-	It can remove 0.001–0.05 mM of imidacloprid and mainly hydrolyzes the C–H bond	[71]
21.	Sodium percarbonate	Artificial neural network and response surface methodology—Box-Behnken design	99.54% of imidacloprid (1 mM) is removed at optimum conditions	[69]
22.	Ti/PbO_2_-Tb	8 mA·cm^−2^ current density, pH 9, temperature 30 °C, and 7.0 g·L^−1^ NaCl electrolyte	76.07% of imidacloprid (150 mg·L^−^^1^) is removed in 5 h; has energy-saving performance and good repeatable use	[70]
23.	Polypyrrole, polyaniline, and sodium alginate composites with peanut husk	pH, pesticide concentration, compound dose, contact time, and temperature	Can effectively remove imidacloprid (25 mg·L^−^^1^)	[64]
24.	Fe_4_O_3_-GO-*β*-CD	Metal organic frameworks	Better improves the adsorption capacity for imidacloprid (100 mg·L^−^^1^)	[75]

**Table 3:** Microbes that degrade imidacloprid.

S. No.	Microorganisms	Isolation Sources	Handling Methods	Results	References
1.	*Hymenobacter latericoloratus* CGMCC 16346	Water sediment	High-performance liquid chromatography (HPLC) and liquid chromatography–mass spectrometry (LC–MS)	Degrades 64.4% of imidacloprid (100 mg·L^−1^) within six days	[134]
2.	*Ochrobactum* sp. BB-1	Tea plantation with long-term application of pesticides	Ultrasonic cell -break method and HPLC	The optimal nitrogen source is 16 g·L^−1^ of yeast extract; the optimal growth conditions include pH 7 and 30 °C	[135]
3.	*Rhodopseudomonas capsulata*	Wastewater	HPLC	Degrades 97% of imidacloprid in five days	[121]
4.	*Acinetobacter* sp. TW	Solid tobacco waste	Morphology, physiological and biochemical tests, Biolog analysis, and 16S rDNA sequencing	Degrades acetamiprid and imidacloprid under broad pH and temperature conditions	[126]
5.	*Sphingomonas* sp. TY	Solid tobacco waste	Morphology, physiological and biochemical tests, Biolog analysis, and 16S rDNA sequencing	Degrades acetamiprid and imidacloprid under broad pH and temperature conditions	[126]
6.	*Pseudomonas* sp. 1G	Neonicotinoid-exposed golf course soil	HPLC and LC-MS	28 °C, microaerophilic	[122]
7.	*Bacillus aerophilus*	Sugarcane field soils	HPLC	Degrades imidacloprid in sandy loam soil	[136]
8.	*Bacillus alkalinitrilicus*	Sugarcane fields	16S rRNA sequence homology and amplification of 16S rRNA gene region	Degrades more than 90% of imidacloprid in 56 days	[130]
9.	*Bradyrhizobiaceae* SG-6C	Soil	HPLC and LC–MS	Hydrolyzes imidacloprid to dechlorinated 6-chloronicotinic acid to 6-hydroxynicotinic acid	[131]
10.	*Ochrobactrum* BCL-1	Tea rhizosphere soil	HPLC	Degrades 78% of imidacloprid within seven days under 30 °C	[129]
11.	*Klebsiella* pneumonia BCH-1	Pesticide-contaminated agricultural field	HPLC	pH 7, 30 °C, static condition	[137]
12.	*Leifsonia* sp. PC-21	Soil	HPLC and LC–MS	Degrades 37–58% of imidacloprid in full strength tryptic soy broth	[114]
13.	*Mycobacterium* sp. MK6	Agricultural soil	HPLC and an enrichment technique	Liquid minimal medium	[133]
14.	*Pseudoxanthomonas indica* CGMCC 6648	Rhizospheric soils	16S rRNA gene analysis, LC–MS, and nuclear magnetic resonance analysis	Different organic acids have substantial effects on olefin imidacloprid production	[123]
15.	*Rhizobium* sp.	Vegetable farming areas	HPLC	Degrades 25.36–45.48% of imidacloprid (25 mg·L^−1^) in limited media	[138])
16.	*Aspergillus terreus* YESM3	Agricultural wastewater	HPLC	Degrades 85% of imidacloprid (25 mg·L^−1^) in Czapek Dox broth medium at pH 4 and 28 °C for 6 days under static conditions	[16]
17.	*Stenotrophomonas* *maltophilia* CGMCC 1.1788	China General Microbiological Culture Collection Center	HPLC	Optimal conditions for the reaction are 30 °C and pH 7.2	[132]
18.	*Pseudomonas* sp. RPT 52	Pesticide-contaminated agricultural field	HPLC	Degrades 46.5% of imidacloprid (0.5 mM) within 40 h; follows first-order kinetics	[120]

**Table 4:** The genes that act on imidacloprid.

S. No.	Genes	Resources	Results	References
1.	*CYP6ER1*	*Nilaparvata lugens*	Cross-resistant; low level of resistance to dinotefuran	[143]
2.	*CYP6FV12*	*Bradysia odoriphaga*	Most expressed in the fourth instar larvae; when the gene is knocked out, mortality increases by 28.62	[97]
3.	*CYP353D1v2*	*Bacillus striatum*	Short-term imidacloprid resistance	[144]
4.	*CYP4C71v2*	*Bacillus striatum*	Long-term imidacloprid resistance	[144]
5.	*CYP4C72*	*Bacillus striatum*	Long-term imidacloprid resistance	[144]
6.	*CYP6AY3v2*	*Bacillus striatum*	Long-term imidacloprid resistance	[144]
7.	*CYP6CM1*	*Bemisia tabaci*	Causes no or a low level of cross-resistance of dinotefuran	[142]
8.	*CYP6BJa/b*	Colorado potato beetle	Mediated by exogenous transcription factors CncC and Maf	[141]
9.	*CYP6BJ1v1*	Colorado potato beetle	Mediated by exogenous transcription factors CncC and Maf	[141]
10.	*CYP9Z25*	Colorado potato beetle	Mediated by exogenous transcription factors CncC and Maf	[141]
11.	*CYP9Z29*	Colorado potato beetle	Mediated by exogenous transcription factors CncC and Maf	[141]
12.	*AChE*	*Diaphorina citri*	RNA interference from acetylcholinesterase	[141]
13.	*ChE*	*Diaphorina citri*	RNA interference from acetylcholinesterase	[141]
14.	*Nlhsp70*	*Nilaparvata lugens*	Full-length, 2805 bp; open reading frame (ORF), 1896 bp	[147]
15.	*UGT375A1*	*Diaphorina citri*	Significantly improves the group resistance	[150]
16.	*UGT383A1*	*Diaphorina citri*	Significantly improves the group resistance	[150]
17.	*UGT383B1*	*Diaphorina citri*	Significantly improves the group resistance	[150]
18.	*UGT384A1*	*Diaphorina citri*	Significantly improves the group resistance	[150]
19.	*Foccα6*	The western flower thrips	Mutations in nicotinic acetylcholine receptor (nAChRs)	[148]
20.	*CPM*	*Rhodopseudomonas capsulata*	Controls the synthesis of P450	[121]
21.	*CYP6A14-1*	*Sitobion avenae* Fabricius	Increases resistance to imidacloprid	[149]
22.	*CYP307A1*	*Sitobion avenae* Fabricius	Increases resistance to imidacloprid	[149]
23.	*GST1-1-1*	*Sitobion avenae* Fabricius	Increases resistance to imidacloprid	[149]
24.	*COE2*	*Sitobion avenae* Fabricius	Increases resistance to imidacloprid	[149]
25.	*CYP4CS3*	*Bemis*ia tabaci	Shows cross resistance to imidacloprid and acetamiprid	[145]
26.	*CYP6CX5*	*Bemisia tabaci*	Shows cross resistance to imidacloprid and acetamiprid	[145]
27.	*CYP6DW2*	*Bemisia tabaci*	Shows cross resistance to imidacloprid and acetamiprid	[145]
28.	*CYP6CM1*	*Bemisia tabaci*	Shows cross resistance to imidacloprid and acetamiprid	[145]
29.	*cch2*	Bradyrhizobiaceae	A candidate gene encoding the initial dechlorination step and is a member of the metal-dependent hydrolase superfamily	[131]

**Table 5:** Proteins that act on imidacloprid.

S. No.	Proteins	Resources	Results	References
1.	ABCG3	*Bemisia tabaci*	Higher expression level in females than in males	[151]
2.	BtCPR	*Bemisia tabaci*	Higher expression level in males and head tissues	[153]
3.	R81T	*Myzus persicae* and aphid cotton	High resistance to imidacloprid; different effects on cyano-nitrosubstituted neonicotinoids and sulfoniloxalic acid	[140]
4.	Aldehyde oxidase	New Zealand rabbits	Plays a more important lethal role than cytochrome P450	[31]
5.	Aldehyde oxidase	Chinese lizard	Key enzymes in nitro reduction	[50]
6.	CYP2C9	Chinese lizard	Key enzymes in nitro reduction	[50]
7.	CYP3A4	Chinese lizard	Hydroxylation and desaturation	[50]
8.	CYP6AY1	*Nilaparvata lugens*	Effectively metabolizes imidacloprid	[154]
9.	CYP6ER1	*Nilaparvata lugens*	Effectively metabolizes imidacloprid	[154]
10.	DcitABC	*Diaphorina citri*	Forty-four DcitABC, expressed in multiple *D. citri* sites	[152]
